# Growth of 3-D flower/grass-like metal oxide nanoarchitectures based on catalyst-assisted oxidation method

**DOI:** 10.1186/1556-276X-9-116

**Published:** 2014-03-13

**Authors:** Lijiao Hu, Yang Ju, Atsushi Hosoi

**Affiliations:** 1 Department of Mechanical Science and Engineering, Nagoya University, Furo-cho, Chikusa-ku, Nagoya 464-8603, Japan

**Keywords:** Flower/grass-like, Nanoarchitecture, Thermal oxidation, Stress gradient, Nickel catalyst

## Abstract

Cu_2_O grass-like and ZnO flower-like nanoarchitectures were fabricated directly on Cu powders and Zn powders using a novel thermal oxidation stress-induced (TOS) method based on catalyst assistance at a low temperature of 150°C under moderate humid atmosphere. The experiments of Al powder were also carried out based on TOS method. Overlapping migration (OLM) of Cu and Zn atoms and toothpaste squeezing migration (TSM) of Al atoms caused by different atom densities in metal oxide materials were studied.

**PACS:**

81. Materials science; 81.07.-b Nanoscale materials and structures: fabrication and characterization; 81.16.Hc Catalytic methods

## Background

Cuprous oxide (Cu_2_O) is a p-type semiconductor metal oxide with a direct band gap of approximately 2.17 eV [1], which has been used as a prospective candidate for low-cost solar energy conversion [2], photocatalysis [3], and sensors [4]. On the other hand, zinc oxide (ZnO) is an n-type semiconductor with a direct band gap of 3.37 eV [5]. Due to its unique optical, electrical, and magnetic properties, it has attracted a great attention and has been widely applied in solar cells and sensors [6-8]. Recently, great efforts have been devoted to fabricate different Cu_2_O and ZnO nanoarchitectures [9-12] because architectures, including geometry, morphology, and hierarchical structures, were found to have a crucial effect on the fundamental properties of micro/nanostructure semiconductors [13-16]. However, until now, all the fabrication methods of Cu_2_O flower-like nanoarchitectures belong to chemical solution methods, which are complex and expensive [17-19]. Moreover, ZnO flower-like nanoarchitectures were fabricated by chemical solution routes and hydrothermal method, which either need highly accurate quantitative chemical materials or need complex experimental steps [20,21].

Recently, we have proposed a novel method using thermal oxidation with participation of catalyst and humidity to fabricate the three-dimensional Cu_2_O flower/grass-like nanoarchitectures (FGLNAs), and the morphology of the Cu_2_O FGLNAs can be controlled by the heating temperature [22]. Although the growth mechanism has been proposed, experimental investigation is still necessary in order to further verify it. Therefore, in this study, Cu powders were used to replace the Cu foil and Cu film for the fabrication of Cu_2_O FGLNAs based on thermal oxidation stress-induced (TOS) method. In addition, to verify the generality of the TOS method, Zn powders were also used to fabricate ZnO FGLNAs. Moreover, to investigate the growth mechanism affected by the atom density of metal oxide materials, Al powders were applied to the TOS method. Based on the experimental results, an overlapping migration (OLM) of Cu and Zn atoms and toothpaste squeezing migration (TSM) of Al atoms due to different atom densities of metal oxide materials were proposed in this study. Compared with the fabrication based on the thermal expansion stress-induced method [23,24], the heating temperature of the approach used here is only 150°C which is at least 200°C lower than that of CuO and 250°C lower than that of ZnO nanowire growth [25]. The experimental fact that metal powders can replace metal foil and film for the growth of FGLNAs further proved that the growth mechanism is based on oxidation extension rather than thermal expansion induced stress.

## Methods

Commercial silicon wafer with a thickness of 0.50 mm was cut into square samples which were 10 × 10 mm^2^ in size. The substrates were then ultrasonically cleaned (Bransonic 1510, Branson Ultrasonics Corp., Danbury, CN, USA) with acetone and washed with ethanol and de-ionized water sequentially to dissolve the contaminations. Afterwards, Ni catalyst was manually daubed on the surface of samples with a cuboid shape having dimensions of 10 × 3 × 2 mm^3^ approximately. The nickel catalyst in this experiment was used as a high temperature resistance electrically conductive coating material (service temperature 538°C Pyro-Duct™ 598-C, Aremco, Inc., Valley Cottage, NY, USA). Cu, Zn, and Al powders were dispersed around Ni on the substrate, respectively. Cu, Zn, and Al powder samples were then heated by a ceramic heater in air atmosphere under 55% to 75% humidity at the temperature of 150°C for 2, 7, and 10 days, respectively.

After the heating process, morphologies of FGLNAs were characterized by scanning electron microscopy (SEM; JSM-7000FK, JEOL Ltd., Akishima, Tokyo, Japan), energy-dispersive X-ray (EDX), and X-ray diffraction (XRD).

## Results and discussion

**Figure 1:**
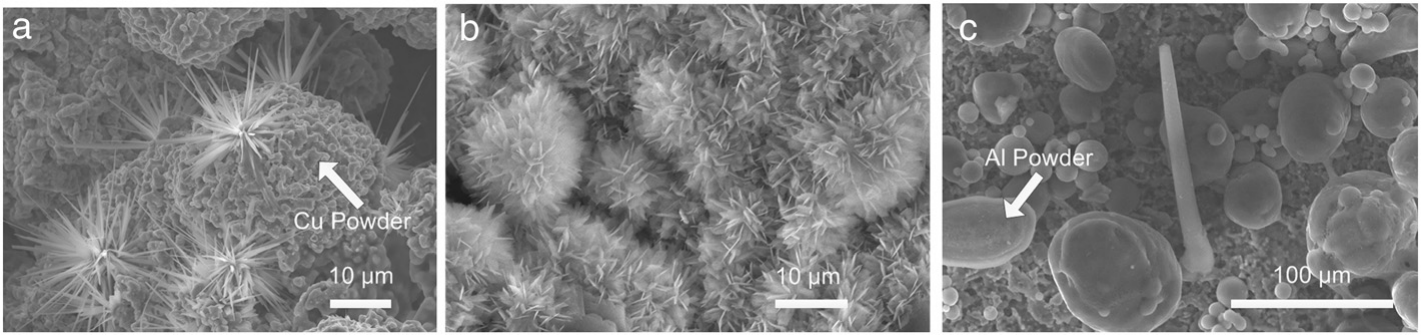
**SEM images. (a)** Cu_2_O grass-like nanoarchitectures, **(b)** ZnO flower-like nanoarchitectures, and **(c)** Al nanowires grown on Cu, Zn, and Al powders at 150°C under 55% to 75% humidity for 2, 7, and 10 days, respectively.

Figure 1 shows SEM images of (a) Cu_2_O grass-like nanoarchitectures, (b) ZnO flower-like nanoarchitectures, and (c) Al nanowires grown on Cu, Zn, and Al powder samples at 150°C under 55% to 75% humidity for 2, 7, and 10 days, respectively. The size of Cu_2_O grass-like nanoarchitectures is 10 to 15 μm, and the width of their petals is 350 to 900 nm. The size of ZnO flower-like nanoarchitectures is 9 to 17 μm, and the width of their petals is 450 to 950 nm. The length of Al nanowires is approximately 120 μm, and the mean diameter is 10 μm approximately. It has been confirmed experimentally that there was no FGLNA growth when the experimental conditions were changed to vacuum environment, without catalyst or under the humidity lower than 55% or higher than 75%, respectively. Therefore, it is thought that besides temperature, oxygen atmosphere, catalyst, and humidity were three essential conditions for the growth of FGLNAs.

**Figure 2:**
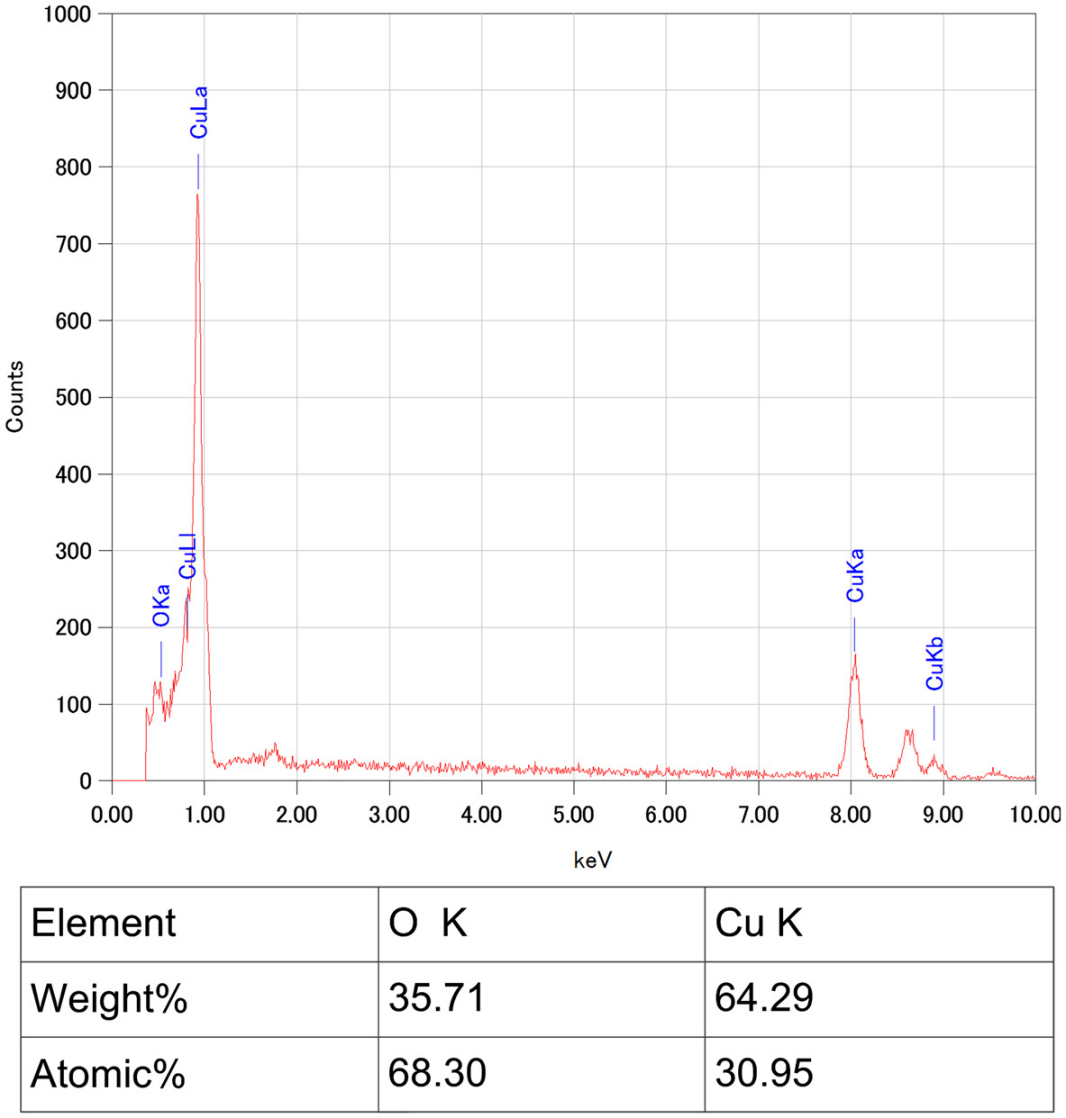
EDX spectra of architectures on Cu powders heated at 150°C under 55% to 75% humidity for 2 days.

**Figure 3:**
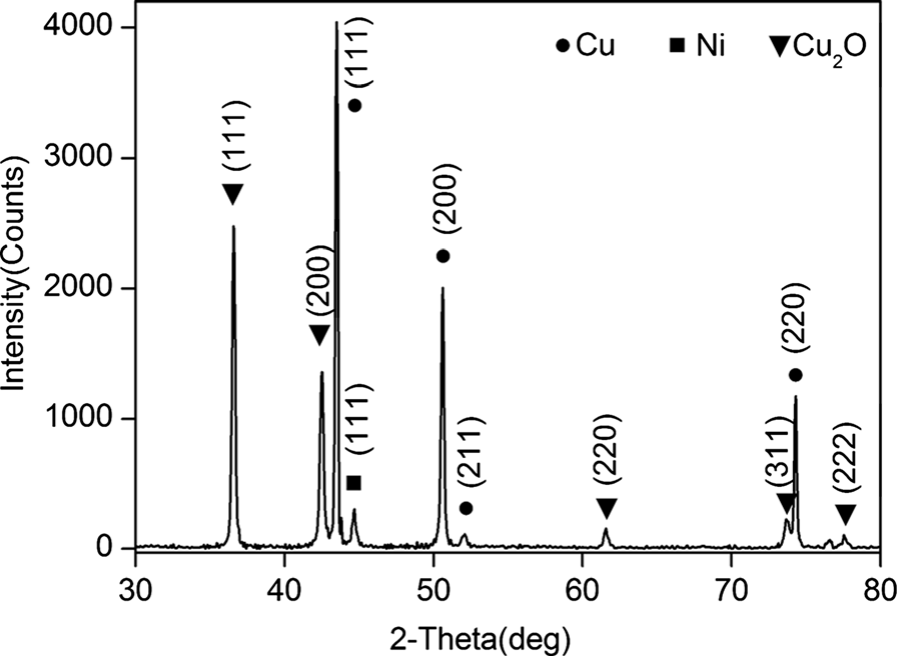
XRD spectra of architectures on Cu powders heated at 150°C under 55% to 75% humidity for 2 days.

**Figure 4:**
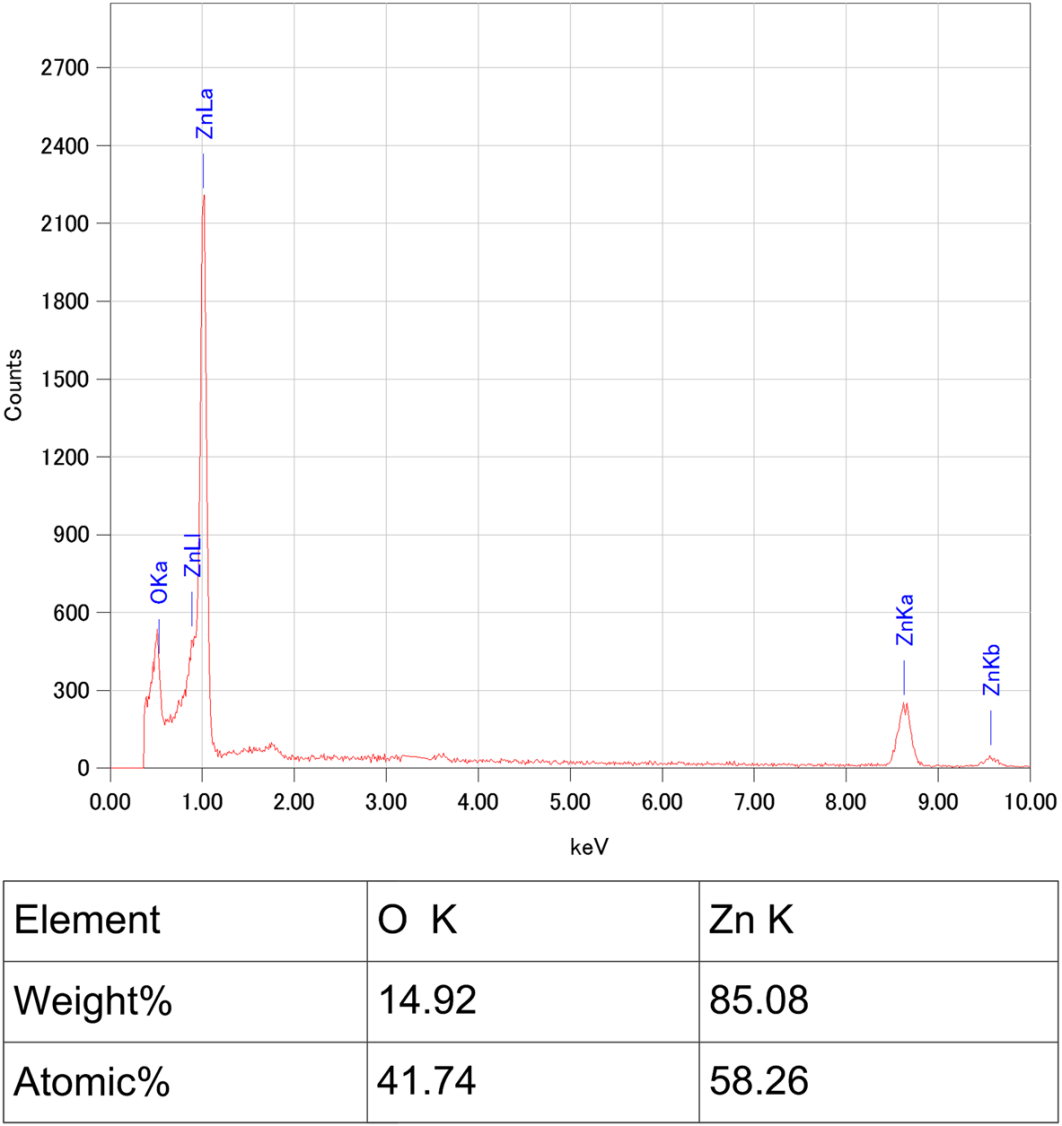
EDX spectra of architectures on Zn powders heated at 150°C under 55% to 75% humidity for 7 days.

**Figure 5:**
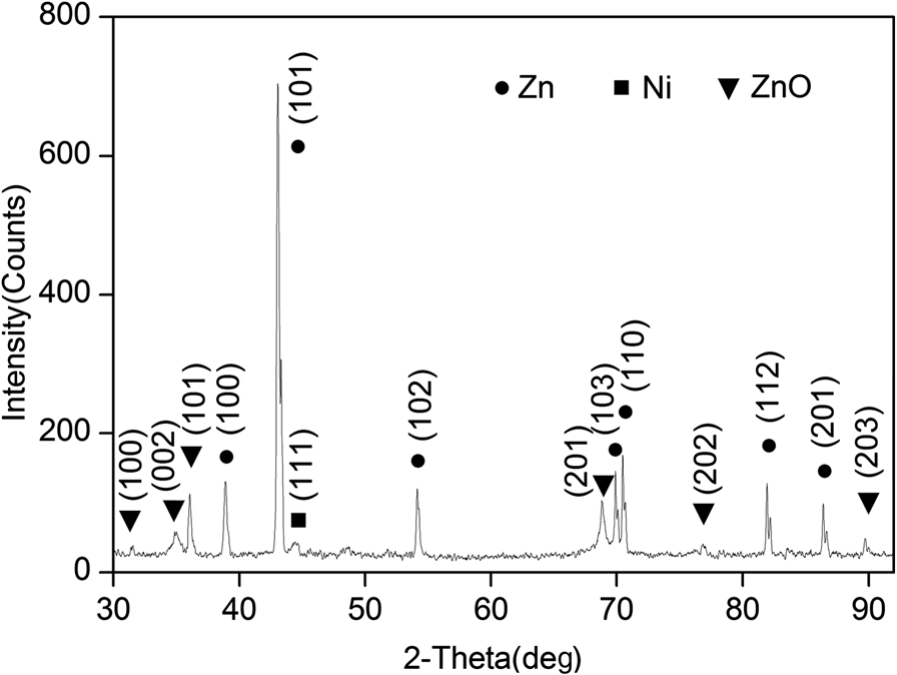
XRD spectra of architectures on Zn powders heated at 150°C under 55% to 75% humidity for 7 days.

**Figure 6:**
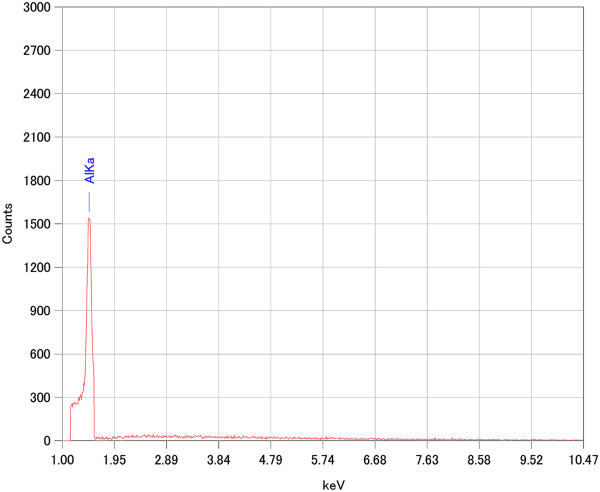
EDX spectra of architectures on Al powders heated at 150°C under 55% to 75% humidity for 10 days.

Figure 2 shows the EDX results of Cu_2_O grass-like nanoarchitectures. It indicates that the grass-like nanoarchitectures are mainly composed of Cu element (30.95%) and oxygen element (68.30%). We also obtained similar EDX results for the other samples. As shown in the XRD spectrum in Figure 3, orientations 111, 200, 311, etc. of Cu_2_O indicate that the FGLNAs are composed of Cu_2_O. As shown in the XRD spectrum, Ni was not oxidized. The reason is that oxidation temperature of Ni is above 400°C. Figure 4 shows EDX analysis of the flower-like nanoarchitectures grown on the Zn powder sample heated at 150°C for 10 days. It indicates that the architectures are mainly composed of Zn element (58.26%) and oxygen element (41.74%). As shown in the XRD spectrum in Figure 5, orientations 002, 101, 201, etc. of ZnO indicate that the FGLNAs are composed of ZnO. As shown in the XRD spectrum, Ni was not oxidized. We also obtained the EDX results of Al nanowires grown on the Al powder sample, as shown in Figure 6. The EDX result indicates that the nanowires grown on the Al powder sample are Al nanowires instead of Al_2_O_3_ nanowires.

**Figure 7:**
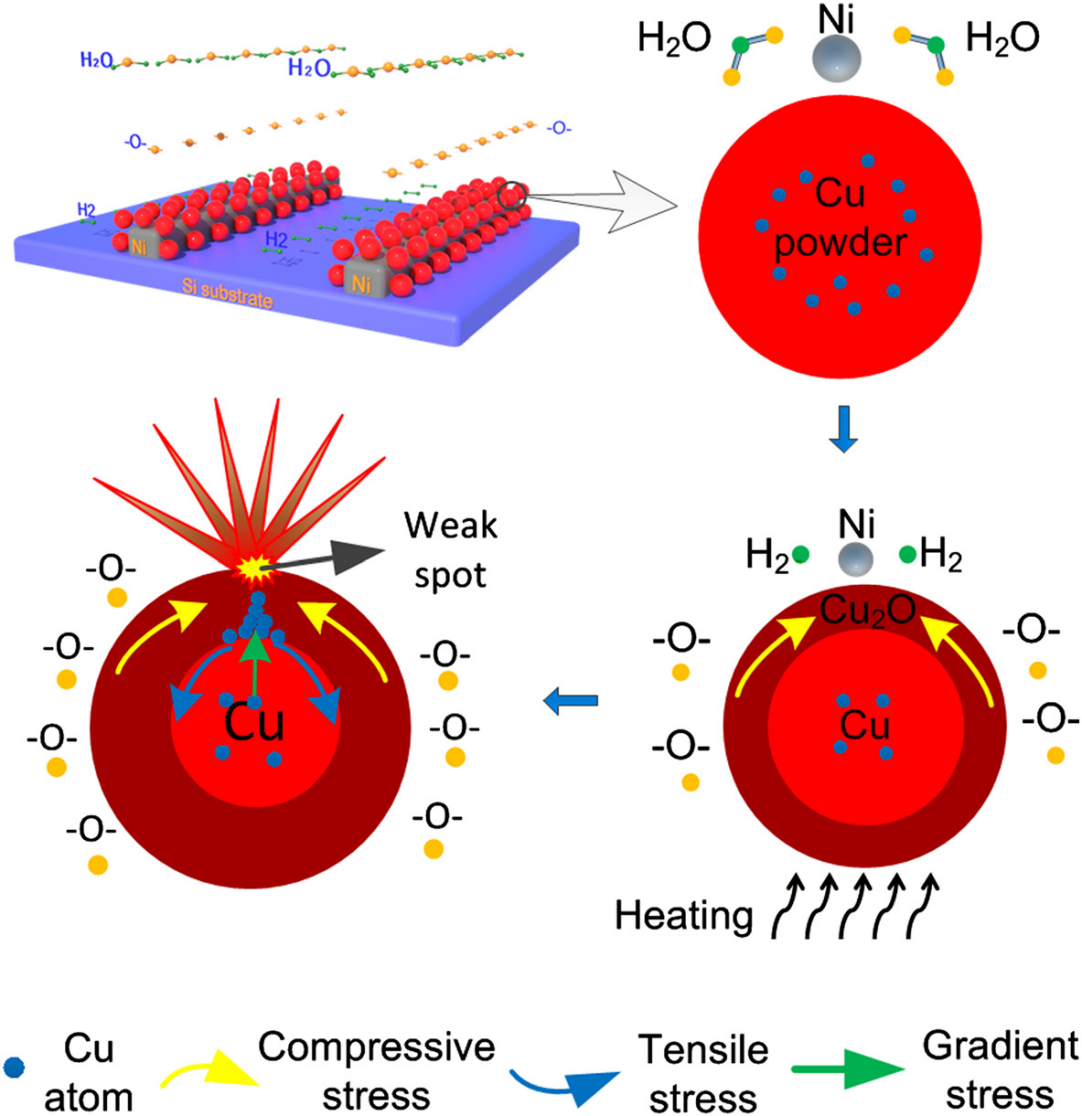
Illustration of grow mechanism.

The growth mechanism is similar with that described in our previous study of Cu foil/film samples [22]. An oxide layer formed on the surface of the metal powders when the samples were heated in air. Taking the Cu powder sample as an example, as shown in Figure 7, stress occurred in the oxide shell due to the oxide volume extension. Stress in the oxide shell can be determined by the Pilling-Bedworth ratio (PBR), which is defined as [26]

(1) $$\text{PBR}=\frac{\text{Volume}\;\text{of}\;\text{oxide}}{\text{Volume}\;\text{of}\;\text{metal}}$$

Here, the volume of metal expresses the consumed metal volume during the formation of oxide shell in the oxidation process. When PBR >1, a compressive stress develops in the oxide shell, while a tensile stress develops in the shell with PBR <1. The larger the difference of PBR from 1, the larger the stress will be. In this experiment, due to volume extension of the Cu_2_O and ZnO oxide shell, the PBR of Cu_2_O and ZnO is larger than 1. Therefore, the Cu_2_O shell suffers tangential compressive stress (TCS) from the core, as illustrated in Figure 7. The same situation happens in the ZnO shell.

Meanwhile, tangential tensile stress (TTS), caused by the reactive force of TCS in the oxide shell, occurred in the inner part of Cu powders at the interface of Cu_2_O (shell)/Cu (core), which leads to the generation of radial stress gradient (RSG) in the thickness direction of the powders. RSG serves as a driving force for the migration of Cu atoms from the center of Cu powders to the interface between the oxide shell and Cu core. At the beginning, the relatively low temperature (150°C) causes relatively lower surface oxidation speed. The Cu_2_O shell which formed on the Cu powder sample is very thin, and the RSG is not large enough. Therefore, the diffused Cu atoms cannot penetrate the oxidation shell. However, sufficient bivalent oxygen ions with two chemical bonds (BOICBs) were generated from the water vapors during the process of hydrogen absorption of nickel catalyst, as indicated in Equation 2.

(2) $$H‒O‒H\overset{\Delta }{\underset{\mathrm{Ni}}{\to }}‒O‒+H_{2}$$

Thus, the low-temperature oxidation was enhanced, and the thickness of the Cu_2_O shell became larger and larger. Therefore, the TCS in the Cu_2_O shell caused by oxide volume extension will be larger than the results without participation of catalyst and humidity, thereby creating larger RSG. On the other hand, the TCS in the oxide shell also made it difficult for Cu atoms to penetrate through the oxide layer from the weak spots on the surface. Consequently, Cu atoms kept accumulating under the oxide shell until there were enough Cu atoms to break the balance, and finally, a large number of Cu atoms suddenly penetrated the oxide shell through the weak spots in a flash. Fewer weak spots appear due to the relatively thicker oxide shell, and a relatively large penetration force is required. A large number of Cu atoms accumulate and penetrate the Cu_2_O shell through the same weak spots. Cu atoms burst out and get more easily oxidized. Moreover, during the formation of FGLNAs, the BOICBs served as a nuclear site. In conclusion, more migrations of Cu atoms caused by larger RSG and larger TCS caused by sufficient BOICBs are two key factors for the growth of FGLNAs.

The mechanism of RSG created in metal powder samples here is different from that in Cu film on Si substrate [10,23,24] in which the gradient stress was generated due to the thermal expansion mismatch of the materials. In this study, RSG was generated due to the TCS and reaction TTS caused by oxide volume extension. However, the experiments in this study proved that oxide volume extension degree has a decisive effect on the growth type of nanostructures. That is the reason that Cu_2_O and ZnO FGLNA growth under a relatively low temperature was realized, instead of that CuO and ZnO nanowires grow under a relatively high temperature.

**Figure 8:**
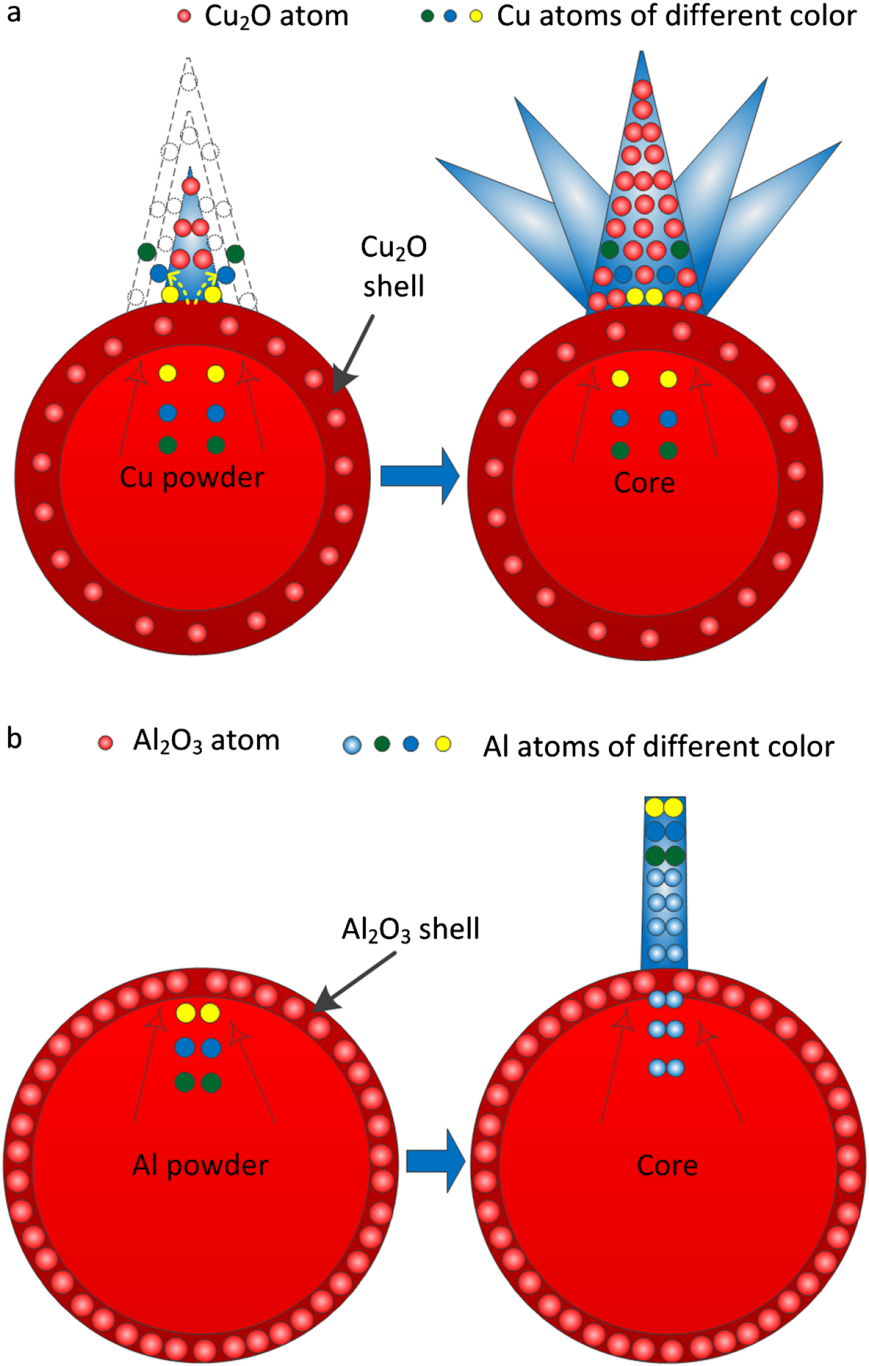
**Illustration of metal atom migration mechanism. (a)** Overlapping migration (OLM) of Cu atoms. **(b)** Toothpaste squeezing migration (TSM) of Al atoms.

To further study the growth mechanism of nanoarchitectures, we focus on the different migration mechanisms of Cu/Zn atoms and Al atoms. It is believed that the migration of Cu/Zn atoms is OLM and the migration of Al atoms is TSM [24]. For the OLM of Cu/Zn atoms, during the growth, the old atoms were covered by the new atoms, which seem to be overlapping. As shown in Figure 8a, using Cu as an example, before penetration through the oxide shell, Cu atoms were ordered by the colors yellow, blue, and green from top to bottom. After the penetration, the sequence of Cu atoms changes reversely. From top to bottom, the Cu atoms are green, blue, and yellow, respectively. On the contrary, the migration of the Al atom is TSM. As shown in Figure 8b, before penetration through the oxide shell, Al atoms were ordered by the colors yellow, blue, and green from top to bottom. After the penetration, the sequence of Al atoms stays the same. In other words, during the penetration, the Al atoms keep the same sequence and the first Al atom that penetrated the oxide shell will migrate to the top of the Al nanowire, which seems like toothpaste squeezing.

The reason is believed to include two aspects. The first is the different atom densities of Cu_2_O, ZnO, and Al_2_O_3_.

(3) $$\rho_{Cu_{2}O}=\frac{m}{V}=6.00\;g/cm^{3}$$

(4) $$\rho_{Cu_{2}O\;\text{molecular}}=\frac{m}{V\cdot M}=0.0416\;\mathrm{mol}/cm^{3}$$

(5) $$\rho_{Cu_{2}O\;\text{atoms}}=\frac{n\cdot m}{V\cdot M}=3\rho_{Cu_{2}O\;\text{molecular}}=0.125\;\mathrm{mol}/cm^{3}$$

Here, *m* is the mass of Cu_2_O, *M* is the molar mass of Cu_2_O, *V* is the volume of Cu_2_O, *n* is the number of atoms in a single molecule (for Cu_2_O, it is 3), $\rho_{Cu_{2}O}$ is the density of Cu_2_O, $\rho_{Cu_{2}O\;\text{molecular}}$ is the Cu_2_O molecular density, and $\rho_{Cu_{2}O\;\text{atoms}}$ is the Cu_2_O atom density. As we know, the density of the Cu_2_O material is 6.00 g/cm^3^, as shown in Equation 3. Cu_2_O molecular density can be calculated to be 0.0416 mol/cm^3^, as shown in Equation 4. Cu_2_O atom density was calculated to be 0.125 mol/cm^3^, as shown in Equation 5. Using the same method, the ZnO and Al_2_O_3_ atom densities are calculated to be 0.138 and 0.194 mol/cm^3^, respectively.

ZnO and Cu_2_O atoms have much lower atom density than Al_2_O_3_ atoms. Taking Cu_2_O as an example, the lower atom density of the Cu_2_O oxide surface layer on FGLNA leaves makes Cu atoms easily penetrate the oxide surface layer and get oxidized. Afterwards, a new oxide layer forms on the top surface layer of FGLNA leaves. As shown in Figure 8a, the yellow Cu atoms are the first Cu atoms to penetrate the oxide surface layer, and after oxidation, the Cu_2_O atoms generated by the yellow Cu atoms would be laying on the bottom layer of Cu_2_O FGLNAs. Due to the sparse Cu_2_O FGLNA oxide surface layer, new blue Cu atoms penetrated the surface layer of FGLNA leaves and get oxidized. As shown in Figure 8a, the yellow dotted line arrow indicates the direction of blue Cu atoms migrating and penetrating the oxide surface layer of FGLNA leaves formed by yellow Cu atoms. Green Cu atoms keep this penetration and oxidation cycle. At last, the layer generated by green Cu atoms lie above the one generated by the blue Cu atoms. Due to this cycle, the leaves of Cu_2_O FGLNAs grow bigger and bigger. For the Al powder case, when the Al powder sample was heated in the air, dense thin oxide layers formed on the surface of Al powders, which prevent atoms from getting further oxidation. As has been calculated above, the Al_2_O_3_ atom density is much higher than Cu_2_O and ZnO atom densities, and this dense oxide shell on the surface of the Al powder makes it difficult for the Al atoms to penetrate through it. Thus, Al atoms keep the same sequence and migrate in a straight line during migration. Therefore, Al nanowires were generated on the surface of the oxide shell. Afterwards, due to the high density of the surface oxide layer on Al nanowires, Al atoms migrate straight and cannot penetrate the surface layer of Al nanowires. Therefore, the present TOS method is unavailable to generate Al_2_O_3_ FGLNAs due to the unique oxidation properties and higher atom density of Al_2_O_3_.

Secondly, according to the previous study, the PBR of Al_2_O_3_ is 1.28 which is much smaller than those of Cu_2_O and ZnO [27]. Higher PBR of Cu_2_O and ZnO means bigger oxide volume extension during oxidation. Therefore, higher TCS and TTS were generated, which results in higher RSG. Higher driving force of RSG leads to more Cu and Zn atoms migrating from the metal core to the interface of the oxide shell. More Cu and Zn atoms accumulate and erupt from the weak spots on the surface of metal powder to form FGLNAs.

**Figure 9:**
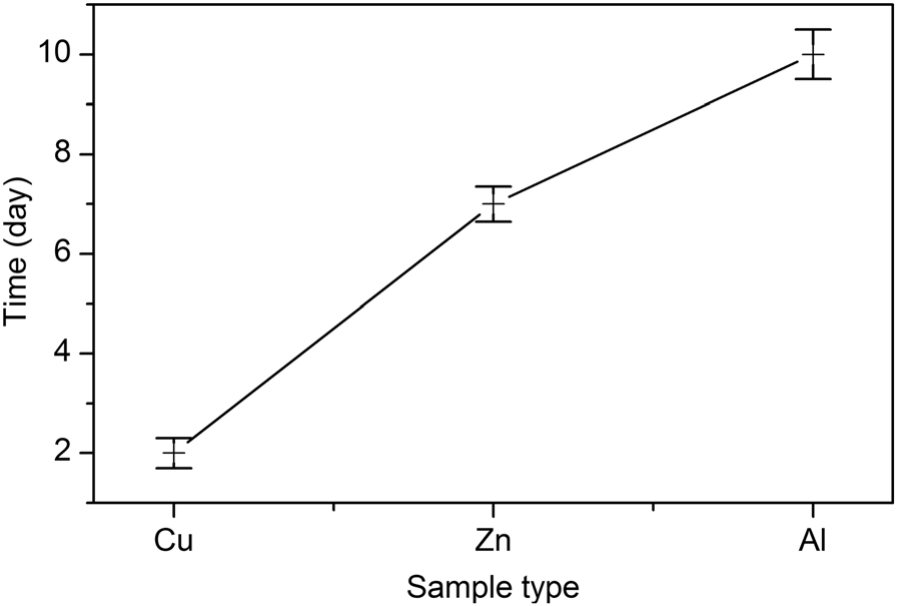
Time required for the first appearance of nanoarchitectures.

On the other hand, the heating time for the first appearance of Cu_2_O, ZnO FGLNAs, and Al nanowires was also observed for the samples of Cu, Zn, and Al powders. As shown in Figure 9, the heating time for the samples of Cu, Zn, and Al powders is 2, 7, and 10 days, respectively. Ranking of PBR from big to small is Cu_2_O, ZnO, and Al_2_O_3_, respectively. Higher PBR leads to higher RSG. Higher RSG promotes the diffusion of Cu atoms, thereby speeding up the growth of FGLNAs. In addition, it is believed that during oxidation, the BOICBs serve as a bridge to connect metal atoms. In the unit cell of Cu_2_O, ZnO, and Al_2_O_3_, BOICBs connect with two Cu atoms, four Zn atoms, and three Al atoms, respectively. Due to the two chemical bonds of BOICBs, it is believed that the combination with two Cu atoms is the easiest. Next is that with four Zn atoms, and the most difficult is that with three Al atoms. Thus, the time required for the first appearance of Cu_2_O, ZnO, and Al nanoarchitectures increases orderly. Moreover, with the same length and width of Ni cuboid, the thickness of the Ni catalyst can also affect the growth time of Cu_2_O FGLNAs, but not their morphology and size. Thinner thickness of the Ni catalyst would lead to longer time for the growth of FGLNAs.

## Conclusions

Cu_2_O and ZnO FGLNAs were successfully fabricated using the TOS method based on catalyst assistance under moderate humid atmosphere. Higher atom density and large oxide volume extension of Cu_2_O and ZnO lead to OLM of Cu and Zn atoms, respectively. On the contrary, smaller atom density and oxide volume extension of Al_2_O_3_ result in TSM of Al atoms. Higher PBR promotes the diffusion of metal atoms, thereby speeding up the growth of FGLNAs. Compared with other methods to fabricate FGLNAs, the TOS method featured remarkable simplicity and cheapness.

## Abbreviations

BOICBs: bivalent oxygen ions with two chemical bonds; FGLNAs: flower/grass-like nanoarchitectures; OLM: overlapping migration; RSG: radial stress gradient; TCS: tangential compressive stress; TOS: thermal oxidation stress-induced; TSM: toothpaste squeezing migration; TTS: tangential tensile stress.

## Competing interests

The authors declare that they have no competing interests.

## Authors' contributions

LJH designed and performed all the experiments, analyzed the data, and wrote the main manuscript text. YJ designed and conducted the whole study. AH helped in the XRD characterization experiments. All authors read and approved the final manuscript.
